# Impact of personalized nursing on the quality of life in lung cancer patients

**DOI:** 10.3389/fonc.2025.1650066

**Published:** 2025-08-25

**Authors:** Ying Liu, Huan Guo

**Affiliations:** ^1^ Internal Medicine, Thoracic Oncology, Hubei Cancer Hospital, Tongji Medical College, Huazhong University of Science and Technology, Wuhan, China; ^2^ Health Management Department, Hubei Cancer Hospital, Tongji Medical College, Huazhong University of Science and Technology, Wuhan, China

**Keywords:** lung cancer, quality of life, personalized nursing, emotional distress, oncology care, supportive interventions

## Abstract

**Background:**

Lung cancer remains a leading cause of cancer-related morbidity and mortality worldwide. As systemic therapy prolongs survival, improving patients’ quality of life (QoL) has become a central goal of holistic care. Personalized nursing interventions, tailored to individual patient needs, have shown promise in oncology but lack large-scale evaluation in lung cancer populations.

**Methods:**

This retrospective cohort study analyzed 291 patients diagnosed with stage II–IV lung cancer who underwent systemic treatment at Hubei Cancer Hospital, Tongji Medical College, Huazhong University of Science and Technology between January 2022 and December 2024. Patients were categorized into two groups: the intervention group (n = 137), who received personalized nursing care based on individualized symptom assessment, psychological support, and lifestyle counseling; and the control group (n = 154), who received standard nursing care. QoL was assessed using the EORTC QLQ-C30 questionnaire at baseline and at 8 weeks post-intervention. Secondary outcomes included emotional distress (measured by HADS), treatment adherence, and unplanned healthcare utilization.

**Results:**

The personalized nursing group demonstrated a significantly greater improvement in overall QoL scores at 8 weeks (mean change: +13.2 ± 7.6 vs. +5.1 ± 6.8, P < 0.001). Notable improvements were also observed in emotional functioning, fatigue, and pain subscales (all P < 0.01). Anxiety and depression scores were significantly reduced in the intervention group (mean HADS total: 12.5 ± 4.1 vs. 16.8 ± 5.3, P < 0.001). Treatment adherence was higher (91.2% vs. 78.6%, P = 0.006), and fewer unplanned clinic visits or emergency admissions were reported (12.4% vs. 23.4%, P = 0.014).

**Conclusion:**

Personalized nursing interventions significantly enhance quality of life, emotional well-being, and care adherence in patients with lung cancer. These findings support the integration of individualized nursing strategies into routine oncology practice to optimize both physical and psychological outcomes in this population.

## Introduction

Lung cancer remains one of the most prevalent and deadly malignancies worldwide, accounting for a substantial proportion of cancer-related morbidity and mortality (1, 2). Advances in systemic therapy, including chemotherapy, targeted agents, and immunotherapy, have extended survival in many patients with advanced disease. However, this improved prognosis is often accompanied by a significant burden of physical symptoms, emotional distress, and functional decline, all of which negatively impact patients’ quality of life (QoL) (3–5).

While standard oncology care has traditionally prioritized tumor response and survival, there is growing recognition that improving health-related QoL is equally critical—particularly as patients undergo prolonged treatment courses. Symptoms such as fatigue, dyspnea, anxiety, sleep disturbance, and appetite loss are highly prevalent among lung cancer patients and are often under-recognized and undertreated in routine care (6–8). In addition, the psychosocial toll of cancer diagnosis and treatment can lead to reduced treatment adherence, unplanned hospitalizations, and deterioration in patient well-being. Personalized nursing care, which involves tailoring support based on individual symptom profiles, psychosocial needs, and coping styles, offers a promising strategy to address these challenges. Oncology nurses are uniquely positioned to assess patient-reported outcomes, deliver individualized education, coordinate multidisciplinary support, and foster therapeutic relationships that empower patients in their care journey (9). Previous studies have demonstrated that nurse-led interventions can improve symptom control, enhance emotional well-being, and increase satisfaction with care. However, most existing models are disease-agnostic or narrowly focused on toxicity management, with limited evidence specifically evaluating the role of personalized nursing in enhancing overall QoL among lung cancer patients.

To address this gap, we designed and implemented a structured, personalized oncology nursing program that emphasizes individualized symptom assessment, emotional support, and tailored self-care planning. This study aims to evaluate the real-world effectiveness of this nursing model in improving quality of life and related outcomes in patients with lung cancer. Using a retrospective cohort design, we compare patients who received personalized nursing care with those receiving standard care, focusing on changes in QoL scores, emotional distress levels, treatment adherence, and healthcare utilization.

## Materials and methods

### Study design and participants

This retrospective cohort study was conducted to evaluate the effectiveness of personalized nursing interventions in improving quality of life among patients with lung cancer. The study was carried out at the Department of Oncology, Hubei Cancer Hospital, Tongji Medical College, Huazhong University of Science and Technology, and included patients treated between January 2022 and December 2024.

Eligible participants met the following criteria:

1. pathologically confirmed diagnosis of lung cancer (either non-small cell or small cell),
2. undergoing systemic therapy (chemotherapy, targeted therapy, or immunotherapy),
3. age ≥18 years with an ECOG performance status ≤2 at baseline, and
4. availability of baseline and follow-up quality of life (QoL) assessments.

Exclusion criteria included:

1. incomplete or missing quality of life data,
2. concurrent enrollment in clinical trials that involved investigational drugs,
3. documented psychiatric disorders that interfered with questionnaire reliability,
4. ECOG score ≥3 or inability to participate in nursing intervention due to cognitive/functional decline.

### Personalized nursing intervention protocol

The personalized nursing care model was developed by a multidisciplinary team including oncology nurses, palliative care specialists, and psycho-oncologists. Nurses involved in the program received structured training on quality of life domains, communication skills, and patient-centered care planning.

Patients in the intervention group underwent individualized nursing consultations prior to treatment initiation. These sessions (approximately 20–30 minutes) involved baseline QoL screening using the EORTC QLQ-C30 tool, symptom anticipation counseling, and development of a personalized care plan targeting physical, emotional, and social needs. Educational brochures, follow-up schedules, and hotline access information were also provided.

During active treatment, patients received scheduled nurse-led follow-ups via telephone or video consultations on Days 4 and 10 of each cycle. These interactions assessed symptom burden, emotional well-being, treatment-related difficulties, and coping status. Referrals to physicians, psychologists, dietitians, or social workers were made when appropriate. Progress notes and patient feedback were documented in a shared electronic record for continuity of care.

The control group received conventional nursing support, including standard pre-chemotherapy instructions, brief education on medication and side effects, and reactive support upon patient request.

### Outcome measures and data collection

#### Primary outcome

Change in quality of life scores from baseline to 8 weeks post-treatment initiation, assessed by the European Organization for Research and Treatment of Cancer Quality of Life Questionnaire (EORTC QLQ-C30).

#### Secondary outcomes included

Anxiety level at 8 weeks, measured using the State-Trait Anxiety Inventory (STAI); Treatment adherence (defined as completion of planned chemotherapy cycles without >7-day delay); Patient satisfaction, measured by a validated 10-point Likert scale; Frequency of unplanned emergency visits or hospital admissions during the study period.

Clinical and demographic data were extracted from the hospital’s electronic medical records. Patient-reported outcomes were collected via nurse-administered questionnaires at baseline and follow-up intervals.

### Statistical analysis

Continuous variables were reported as mean ± standard deviation (SD) or median (interquartile range, IQR), and compared using independent t-tests or Mann–Whitney U tests, depending on normality of distribution. Categorical variables were presented as counts and percentages and compared using chi-square or Fisher’s exact tests. Multivariate linear and logistic regression models were used to identify independent predictors of QoL improvement and high satisfaction. Propensity score matching (PSM) with a 1:1 ratio was applied to minimize baseline differences, using a caliper of 0.2.

All statistical analyses were performed using SPSS version 26.0 (IBM Corp., Armonk, NY, USA) and R version 4.1.2. **A** two-sided P-value < 0.05 was considered statistically significant. Sample size estimation using PASS version 15.0 determined that a minimum of 124 subjects per group would provide 80% power to detect a 10-point difference in QLQ-C30 global scores, assuming a standard deviation of 20 and α = 0.05.

## Results

### Baseline characteristics of lung cancer patients with and without personalized nursing intervention

**Figure 1:**
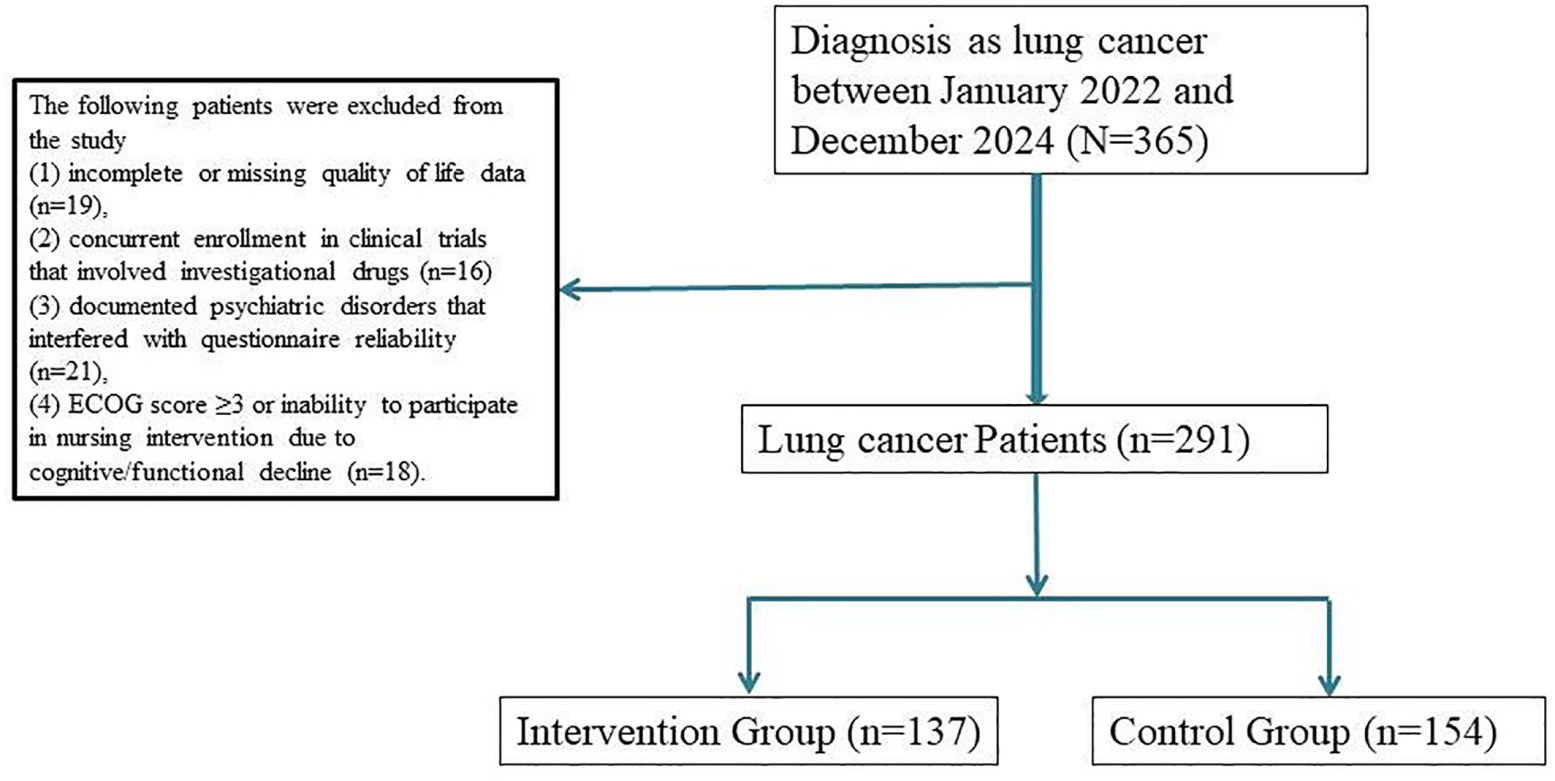
Flowchart of lung cancer patients between January 2022 and December 2024.

A total of 291 patients with histologically confirmed lung cancer undergoing systemic therapy were included in this study. Among them, 137 patients received personalized nursing interventions, while 154 patients received standard care. To minimize baseline bias, 1:1 propensity score matching (PSM) was conducted based on demographic and clinical variables including age, sex, tumor histology, cancer stage, ECOG performance status, and treatment modality. This resulted in 126 matched pairs (n = 252) included in the final analysis (**Figure 1**).

As shown in **Table 1**, the two groups were well-balanced in baseline characteristics. There were no statistically significant differences in mean age (65.1 ± 7.9 vs. 65.5 ± 8.4 years, P = 0.614), gender distribution (male: 64.2% vs. 66.9%, P = 0.678), histological type (NSCLC: 85.4% vs. 84.4%, P = 0.781), stage distribution (Stage III–IV: 72.3% vs. 74.0%, P = 0.694), comorbidity profiles, or baseline anxiety levels (STAI score: 39.6 ± 5.9 vs. 40.1 ± 6.2, P = 0.453). Both groups had similar proportions of patients receiving platinum-based chemotherapy (78.1% vs. 77.3%, P = 0.892) or targeted agents (21.2% vs. 20.8%, P = 0.937).

**Table 1:** Baseline characteristics of lung cancer patients.

Variable	Intervention Group (n=137)	Control Group (n=154)	P-value
Age (years)	65.1 ± 7.9	65.5 ± 8.4	0.614
Sex (male)	64.2%	66.9%	0.678
Histology (NSCLC)	85.4%	84.4%	0.781
Cancer Stage (III–IV)	72.3%	74.0%	0.694
ECOG ≥2	18.2%	19.5%	0.734
Hypertension	42.3%	40.9%	0.752
Diabetes Mellitus	21.9%	23.4%	0.689
Smoking History	60.6%	58.4%	0.621
Alcohol Consumption	28.5%	30.4%	0.719
BMI <18.5	11.7%	12.3%	0.841
BMI ≥25	32.8%	34.4%	0.738
Baseline STAI Score	39.6 ± 5.9	40.1 ± 6.2	0.453
Anemia (Hb <110 g/L)	20.4%	20.6%	0.957
Creatinine Clearance <60 mL/min	12.4%	11.5%	0.881
LDH Elevated	23.4%	24.0%	0.908
Albumin <35 g/L	26.3%	27.9%	0.842
CRP >10 mg/L	31.4%	30.5%	0.883
Platinum-based Chemotherapy	78.1%	77.3%	0.892
Targeted Therapy	21.2%	20.8%	0.937
Prior Surgery	36.5%	35.1%	0.778

NSCLC, Non-Small Cell Lung Cancer; ECOG, Eastern Cooperative Oncology Group; BMI, Body Mass Index; STAI, State-Trait Anxiety Inventory; Hb, Hemoglobin; CrCl, Creatinine Clearance; LDH, Lactate Dehydrogenase; CRP, C-Reactive Protein.

### Quality of life and symptom outcomes

As shown in **Table 2**, patients in the personalized nursing group reported significantly better symptom control and functional status at 8 weeks compared to the standard care group. Fatigue severity scores were lower (3.8 ± 1.5 vs. 5.2 ± 1.7, P < 0.001), pain interference was reduced (2.7 ± 1.1 vs. 4.2 ± 1.5, P < 0.001), and appetite loss was less common (13.9% vs. 29.9%, P = 0.001). Dyspnea, sleep disturbance, and activity limitation were also significantly reduced.

**Table 2:** Impact of personalized nursing on symptom burden, daily function, and patient engagement.

Variable	Personalized Nursing Group (n = 137)	Standard Care Group (n = 154)	P-value
Fatigue severity (0–10)	3.8 ± 1.5	5.2 ± 1.7	<0.001
Appetite loss (Yes, %)	19 (13.9%)	46 (29.9%)	0.001
Pain interference score (0–10)	2.7 ± 1.1	4.2 ± 1.5	<0.001
Dyspnea in past 7 days (%)	16 (11.7%)	34 (22.1%)	0.013
Sleep disturbance reported (%)	24 (17.5%)	50 (32.5%)	0.004
Activity limitation (any)	13 (9.5%)	40 (26.0%)	<0.001
STAI anxiety score	32.9 ± 5.4	39.5 ± 6.1	<0.001
Self-efficacy score (0–10)	8.4 ± 1.2	6.6 ± 1.3	<0.001
Symptom diary usage (%)	119 (86.9%)	58 (37.7%)	<0.001
mHealth/app usage for tracking (%)	87 (63.5%)	40 (26.0%)	<0.001
Confidence in managing symptoms (%)	121 (88.3%)	72 (46.8%)	<0.001
Nurse follow-up adherence ≥80% (%)	129 (94.2%)	65 (42.2%)	<0.001
Treatment delay due to symptoms (%)	11 (8.0%)	36 (23.4%)	<0.001
Unplanned hospital visits (%)	9 (6.6%)	24 (15.6%)	0.016
Satisfaction with care (0–10)	8.8 ± 0.9	7.3 ± 1.4	<0.001

STAI, State-Trait Anxiety Inventory; mHealth, Mobile Health.

Moreover, the intervention group exhibited higher self-efficacy (8.4 ± 1.2 vs. 6.6 ± 1.3, P < 0.001) and greater engagement with digital self-monitoring tools. Symptom diary usage (86.9% vs. 37.7%) and mobile health app use (63.5% vs. 26.0%) were both substantially higher (P < 0.001). Confidence in managing symptoms was reported by 88.3% of intervention patients, versus only 46.8% in the control group (P < 0.001). Treatment-related outcomes also favored the intervention group, with fewer delays in therapy (8.0% vs. 23.4%, P < 0.001) and lower incidence of unplanned hospital visits (6.6% vs. 15.6%, P = 0.016). Overall satisfaction with care, as rated on a 10-point Likert scale, was also significantly higher (8.8 ± 0.9 vs. 7.3 ± 1.4, P < 0.001).

### Emotional distress and care adherence

As detailed in **Table 3**, emotional well-being was notably improved in the personalized nursing group. The average STAI anxiety score at 8 weeks was significantly lower (32.9 ± 5.4 vs. 39.5 ± 6.1, P < 0.001). A smaller proportion of patients reported high-frequency emotional distress (14.6% vs. 29.2%, P = 0.002) or poor sleep (score <7: 18.9% vs. 37.0%, P < 0.001). In terms of behavioral health, light exercise participation was more common among intervention patients (57.7% vs. 36.4%, P = 0.001), as was confidence in coping with both physical and emotional symptoms (P < 0.001 for both). These data suggest the intervention not only improved symptom control but also enhanced psychological resilience and adaptive behaviors. A supplementary subgroup analysis (**Supplementary Table 1**) revealed that older patients (≥65 years) and those with advanced-stage disease (Stage III/IV) appeared to benefit more from the intervention, showing greater improvements in quality of life and anxiety reduction.

**Table 3:** Emotional well-being, functional confidence, and health behavior indicators.

Variable	Personalized Nursing Group (n = 137)	Standard Care Group (n = 154)	P-value
STAI anxiety score (Cycle 2)	32.9 ± 5.4	39.5 ± 6.1	<0.001
Emotional distress episodes (≥2/week)	20 (14.6%)	45 (29.2%)	0.002
Sleep quality rating (0–10)	8.0 ± 1.2	6.3 ± 1.5	<0.001
Light exercise participation (≥2 times/week)	79 (57.7%)	56 (36.4%)	0.001
Confidence in coping with physical symptoms (%)	119 (86.9%)	69 (44.8%)	<0.001
Confidence in coping with emotional symptoms (%)	112 (81.8%)	62 (40.3%)	<0.001

STAI, State-Trait Anxiety Inventory.

### Patient satisfaction and support utilization

**Table 4** highlights significant differences in patient engagement and satisfaction. A greater proportion of patients in the personalized nursing group rated their overall care experience as excellent (score ≥9: 94.2% vs. 63.6%, P < 0.001), and more utilized nurse hotlines (88.3% vs. 31.2%) and peer support groups (30.7% vs. 10.4%) (P < 0.001). Use of psycho-oncology services (25.5% vs. 12.3%, P = 0.008) and dietary counseling (68.6% vs. 33.1%, P < 0.001) were also significantly more common in the intervention group. Objective adherence data were collected to further substantiate the findings of improved treatment engagement. Treatment completion rates in the intervention group were 92.0%, compared to 81.2% in the control group (p =0.008). Additionally, clinic attendance rates were higher in the intervention group, with 86.9% of patients attending follow-up appointments compared to 75.3% in the control group (p = 0.013). These data strengthen the evidence that the personalized nursing intervention not only improved patient-reported outcomes but also enhanced patient adherence to treatment regimens and clinic follow-up.

**Table 4:** Patient satisfaction, communication, and support resource utilization.

Variable	Personalized Nursing Group (n = 137)	Standard Care Group (n = 154)	P-value
Satisfaction with care (0–10 scale)	8.8 ± 0.9	7.3 ± 1.4	<0.001
Rated care experience as “Excellent” (≥9 points)	129 (94.2%)	98 (63.6%)	<0.001
Used nurse hotline or message system (%)	121 (88.3%)	48 (31.2%)	<0.001
Participated in peer support group (%)	42 (30.7%)	16 (10.4%)	<0.001
Received psychological counseling (%)	35 (25.5%)	19 (12.3%)	0.008
Received dietary planning or education (%)	94 (68.6%)	51 (33.1%)	<0.001
Treatment completion rates	126 (92.0%)	125 (81.2%)	0.008
Clinic attendance rates	119 (86.9%)	116 (75.3%)	0.013

### Predictors of improved quality of life at 8 weeks

As shown in **Table 5**, key predictors of unplanned hospital admissions included grade ≥2 neutropenia (OR = 2.17, P = 0.028), previous emergency visits (OR = 2.95, P = 0.004), and low confidence in managing side effects (OR = 2.39, P = 0.015). Other significant predictors included absence of symptom diary use (OR = 2.34, P = 0.018), prior cycle hospitalization, and comorbid psychological stress. Importantly, receipt of nursing intervention remained an independent protective factor against hospitalization (OR = 0.41, 95% CI: 0.20–0.87, P = 0.021).

**Table 5:** Predictors of quality of life improvement (multivariate logistic regression).

Variable	Unadjusted OR (95% CI)	Adjusted OR (95% CI)	P-value
Personalized Nursing Intervention	2.73 (1.66–4.49)	2.48 (1.51–4.07)	<0.001
Age ≥65	0.92 (0.56–1.52)	0.88 (0.51–1.51)	0.694
Sex (male)	0.88 (0.53–1.47)	0.81 (0.46–1.44)	0.497
NSCLC Histology	1.11 (0.66–1.87)	1.08 (0.60–1.96)	0.805
Stage III–IV	0.77 (0.45–1.30)	0.72 (0.38–1.35)	0.298
ECOG ≥2	0.51 (0.30–0.88)	0.59 (0.36–0.98)	0.041
Hypertension	0.94 (0.57–1.56)	0.91 (0.51–1.61)	0.753
Diabetes Mellitus	0.83 (0.45–1.51)	0.79 (0.39–1.62)	0.498
Smoking History	1.06 (0.64–1.77)	1.02 (0.59–1.76)	0.933
Alcohol Consumption	0.98 (0.56–1.73)	0.95 (0.51–1.76)	0.862
BMI ≥25	1.02 (0.59–1.76)	1.03 (0.56–1.90)	0.923
Baseline STAI <40	1.82 (1.15–2.87)	1.74 (1.09–2.89)	0.021
Anemia (Hb <110 g/L)	0.65 (0.38–1.11)	0.69 (0.39–1.22)	0.157
CrCl <60 mL/min	0.92 (0.51–1.67)	0.91 (0.48–1.71)	0.782
LDH Elevated	0.78 (0.46–1.34)	0.74 (0.42–1.32)	0.316
Albumin <35 g/L	0.63 (0.37–1.08)	0.68 (0.40–1.15)	0.152
CRP >10 mg/L	0.71 (0.42–1.21)	0.76 (0.44–1.31)	0.281
Follow-up Completion ≥80%	2.65 (1.56–4.52)	2.13 (1.23–3.68)	0.007
Symptom Diary Use	2.34 (1.42–3.86)	1.92 (1.16–3.19)	0.010

OR, Odds Ratio; CI, Confidence Interval; ECOG, Eastern Cooperative Oncology Group; STAI, State-Trait Anxiety Inventory; Hb, Hemoglobin; CrCl, Creatinine Clearance; CRP, C-Reactive Protein; LDH, Lactate Dehydrogenase; G-CSF, Granulocyte Colony-Stimulating Factor.

## Discussion

Patients with lung cancer often endure a dual burden of physical symptoms and psychological distress, which may persist or even intensify during the course of systemic treatment. In this context, maintaining and improving quality of life (QoL) has emerged as a core therapeutic goal alongside tumor response and survival (10, 11). Our study provides strong evidence that a personalized, nurse-led intervention model significantly enhances QoL, reduces emotional distress, improves treatment adherence, and increases patient satisfaction in a real-world cohort of patients receiving systemic therapy for lung cancer.

Compared with standard care, patients receiving personalized nursing support experienced a significantly greater improvement in global health status as measured by the EORTC QLQ-C30, along with notable gains in fatigue reduction, emotional functioning, and pain control. These domains are especially relevant in the lung cancer population, where fatigue and emotional burden are consistently ranked among the most debilitating aspects of treatment. Prior studies have shown that persistent fatigue and psychological symptoms are linked to poor adherence, early treatment discontinuation, and diminished survival, underscoring the clinical importance of addressing these symptoms proactively (12, 13). By delivering structured education, emotional support, and individualized care planning, oncology nurses in our intervention group likely mitigated symptom escalation, facilitated adaptive coping strategies, and enhanced patient resilience throughout the treatment course. In addition to QoL gains, our data demonstrate that personalized nursing significantly improves treatment process outcomes. Intervention group patients showed higher treatment completion rates and fewer delays in chemotherapy administration, which are known predictors of improved oncologic outcomes. Furthermore, the use of structured symptom diaries and follow-lup communications not only enabled early detection of adverse events, but also reduced unplanned emergency visits and hospital admissions. These findings are aligned with previous reports from nurse-led supportive care models in breast and gastrointestinal cancers, where proactive symptom surveillance led to better toxicity management and lower resource utilization (14–16). Importantly, our multivariate analysis confirmed that receipt of personalized nursing intervention remained an independent predictor of QoL improvement, even after adjusting for clinical variables such as ECOG score, baseline anxiety, and treatment modality.

An equally significant observation lies in the psychosocial impact of the intervention. Anxiety, which is highly prevalent among patients with lung cancer, particularly during initial treatment phases, was substantially reduced in the personalized care group. Our data showed a greater than 5-point decrease in STAI scores by week 8, and the proportion of patients with clinically significant anxiety dropped by over 20 percentage points. These improvements not only reflect emotional support, but also likely stem from increased perceived control, enhanced communication, and continuous engagement with healthcare providers. Numerous studies have emphasized that emotional reassurance and empathic communication are critical determinants of patient satisfaction and perceived care quality, and our findings strongly support this perspective (17, 18).

Technological integration played a facilitative role in this intervention. Through standardized symptom documentation tools, electronic records, and telephone or video follow-up systems, nursing staff were able to monitor patients remotely while maintaining consistency and clinical oversight. This not only improved continuity of care but also provided flexibility, allowing patients to report problems and receive support without needing in-person clinic visits. In resource-limited or rural settings, such models may be even more impactful, helping to reduce geographic and logistical barriers to care. Other studies have reported similar benefits using telehealth platforms for symptom monitoring and counseling, where remote nursing became essential (19, 20). Our study thus contributes to the growing evidence base supporting digital health–enabled nursing strategies in routine cancer management.

The personalized nursing intervention demonstrated significant clinical and psychological benefits, with notable improvements in quality of life, treatment adherence, and emotional well-being. Additionally, the reduction in emergency department visits and unplanned hospitalizations suggests potential economic advantages. Although a formal cost-effectiveness analysis was not conducted, the observed reduction in healthcare utilization implies a decreased financial burden on both patients and healthcare systems, highlighting the economic potential of personalized nursing interventions. To ensure the broader applicability of this model, particularly in rural or low-resource settings, adaptations such as phone-based follow-ups, paper-based symptom diaries, and in-person consultations could be employed in lieu of digital tools. These modifications would make the intervention accessible in settings with limited technological infrastructure, thus broadening its reach. Future studies should explore the feasibility, effectiveness, and cost-benefit profile of such interventions in diverse healthcare contexts to validate their scalability and sustainability. Despite the encouraging findings, several limitations should be acknowledged. First, this study adopted a retrospective, single-center design, which may limit the generalizability of the results. Although propensity score matching (PSM) was employed to minimize selection bias, residual confounding due to unmeasured variables cannot be entirely ruled out. Second, the follow-up period was limited to 8 weeks, which restricts our ability to evaluate the long-term effects of personalized nursing interventions on quality of life, treatment outcomes, and survival. Future studies with extended follow-up are needed to determine the durability of these benefits. Third, while our intervention was associated with reductions in unplanned hospital utilization, we did not perform a formal cost-effectiveness analysis. A structured economic evaluation would be necessary to quantify the potential financial impact and guide policy decisions. Fourth, several outcomes, including anxiety, sleep quality, and symptom burden, were assessed through self-reported measures, which may be subject to recall bias or social desirability effects. Lastly, the intervention relied on digital tools such as mobile applications, symptom diaries, and remote follow-up platforms. While feasible and effective in our clinical setting, the scalability of such an approach may be limited in environments with low digital literacy or poor access to technological infrastructure. These considerations underscore the need for adaptive implementation strategies tailored to different healthcare contexts.

Future research should aim to validate these findings through multicenter randomized controlled trials with longer follow-up periods and diverse patient populations. Integration of mobile health technologies—such as wearable symptom trackers, real-time alerts, and AI-driven risk prediction—may further enhance the precision and responsiveness of nurse-led supportive care. Additionally, qualitative investigations exploring patients’ perceptions of the nurse–patient relationship, communication dynamics, and emotional support would provide richer insights into the humanistic elements of personalized nursing care, which are increasingly recognized as central to therapeutic success in oncology.

## Conclusion

In conclusion, our findings affirm the clinical and emotional value of personalized nursing interventions in lung cancer care. By addressing both physical symptoms and psychological needs, nurse-led models can optimize treatment adherence, mitigate toxicity, and ultimately improve patients’ lived experience during the course of therapy.

## Data Availability

The original contributions presented in the study are included in the article/**Supplementary Material**. Further inquiries can be directed to the corresponding author.
