# Supplementary material for: Impact of personalized nursing on the quality of life in lung cancer patients
Source: Front Oncol. 2025 Aug 25;15:1650066. doi: 10.3389/fonc.2025.1650066 (PMC12415023; doi:10.3389/fonc.2025.1650066)
Supplement: Supplementary file 1 [file Table1.docx]

Supplementary Table 1. Subgroup Analysis of Quality of Life Improvement and Anxiety Reduction.

| Subgroup | QoL Improvement (EORTC QLQ-C30) | Anxiety Reduction (STAI) | p-value (QoL) | p-value (Anxiety) |
| --- | --- | --- | --- | --- |
| Age < 65 | +15% | –18% | – | – |
| Age ≥ 65 | +35% | –22% | 0.03 | – |
| Stage I/II | +20% | –15% | – | – |
| Stage III/IV | +22% | –40% | – | 0.01 |

Abbreviations: QoL – Quality of Life; STAI – State-Trait Anxiety Inventory; EORTC QLQ-C30 – European Organisation for Research and Treatment of Cancer Quality of Life Questionnaire
